# Efficacy of Preventing Relapse Evaluated by a Multicenter Randomized Double-Blind Placebo-Controlled Withdrawal Study of Escitalopram in Japanese Adolescents with Major Depressive Disorder

**DOI:** 10.1089/cap.2023.0048

**Published:** 2023-12-15

**Authors:** Takuya Saito, Hidetoshi Takahashi, Noa Tsujii, Tsuyoshi Sasaki, Yuta Yamaguchi, Masahiro Takatsu, Masaki Sato

**Affiliations:** ^1^ Department of Child and Adolescent Psychiatry, Hokkaido University Hospital, Sapporo, Japan.; ^2^ Kochi Medical School Department of Child and Adolescent Psychiatry, Kochi University, Kochi, Japan.; ^3^ Department of Child Mental Health and Development, Toyama University Hospital, Toyama, Japan.; ^4^ Department of Child Psychiatry, Chiba University Hospital, Chiba, Japan.; ^5^ Mochida Pharmaceutical Co., LTD., Shinjuku-ku, Japan.

**Keywords:** major depressive disorder, escitalopram, preventing relapse, adolescents, Japan

## Abstract

**Objective:**

To evaluate the efficacy and safety of escitalopram (ESC) in a 48-week relapse prevention study in Japanese adolescents with major depressive disorder (MDD).

**Methods:**

This was a 48-week multicenter randomized double-blind placebo-controlled parallel-group study of patients aged 12–17 years with MDD. Patients received ESC for 12 weeks as an open-label treatment period (open-label period). Patients who achieved criteria for remission or response in the open-label period received either ESC or placebo for 36 weeks as a double-blind treatment period (double-blind period). The primary endpoint was the time to relapse during the double-blind period. Safety was evaluated in terms of type, incidence, and severity of adverse events.

**Results:**

Of the 128 patients who entered the open-label period, 80 patients entered the double-blind period, all of whom were in the primary analysis population. The primary endpoint, time to relapse, was marginally less than statistically significant between the ESC and placebo groups (*p* = 0.051, log-rank test). In the Cox proportional hazards model, the estimated hazard ratio [two-sided 95% confidence interval] for relapse in the placebo group versus the ESC group was 2.96 [0.94, 9.30]. There were statistically significant differences between the ESC and placebo groups in several secondary endpoints (change in Children's Depression Rating Scale-Revised, change in Clinical Global Impressions-Severity Scale, etc.). No notable safety/tolerability issues were observed in this study compared with the results of studies in Japanese adults with MDD.

**Conclusions:**

Superiority of ESC over placebo for relapse prevention in Japanese adolescents aged 12–17 years with MDD could not be verified with time to relapse evaluated by log-rank test. However, secondary endpoint results and a *post hoc* analysis of time to relapse suggest that ESC may be effective in preventing MDD relapse. No notable safety/tolerability issues were observed compared with the results of studies in Japanese adults with MDD. Study Registry Number: jRCT2080224520

## Introduction

It was previously thought that major depressive disorder (MDD), as diagnosed by the criteria used in adults, did not occur in pediatric patients. However, it became clear in the late 1970s that MDD satisfying adults diagnostic criteria exist even in adolescents (Puig et al., 1978). A meta-analysis reported the prevalence of MDD in children (<13 years) to be 2.8% and in adolescents (13–18 years) to be 5.6% (Costello et al., 2006).

Manifestations of MDD in pediatric patients have been reported to vary with developmental stage, with clinical symptoms beginning to resemble those of adults with increasing age (Carlson and Kashani, 1988). Although the prognosis of MDD in pediatric patients often improves over a period of 1–2 years, there are many cases of subsequent relapse (Fombonne et al., 2001).

Two main approaches are available for the treatment of MDD in pediatric patients: psychoeducational intervention, such as psychotherapy, and pharmacological therapy. For mild depression, according to the National Guidelines for the Treatment of MDD in Japan (Japanese Society of Mood Disorders, 2016), and the UK NICE guidelines (National Institute for Health and Care Excellence, 2019), a follow-up for a certain period of time is recommended, with provision of psychoeducation and environmental adjustment at home and school.

For moderate and severe depression, psychotherapy or pharmacological therapy are also recommended. In the United States, fluoxetine (aged 8–18 years), and escitalopram (ESC; aged 12–17 years) have been approved as pharmacological therapies for pediatric MDD patient by the Food and Drug Administration (FDA). No pharmacological therapies have been approved for use in pediatric patients in Japan. Therefore, pharmacological therapy for pediatric MDD patients in Japan appears to be an unmet medical need.

ESC is a serotonin reuptake inhibitor developed by H. Lundbeck A/S. It has been shown to be effective in adult patients with MDD worldwide, including Japan. A double-blind placebo-controlled study in pediatric MDD patients aged 6–17 years was conducted in the United States, and *post hoc* analyses in adolescents with depression (12–17 years) suggested that ESC was more effective than placebo in improving depressive symptoms (Wagner et al., 2006).

This was followed by a double-blind placebo-controlled study of MDD adolescent patients aged 12–17 years, which showed statistically significant differences from placebo (Emslie et al., 2009). ESC was approved in 2009 in the United States for the treatment of MDD in adolescent patients aged 12–17 years.

As mentioned earlier, relapse is common in MDD. Therefore, it is important to investigate the efficacy of antidepressants not only in the acute phase, but also in the prevention of relapse and recurrence after symptomatic remission or response. Since the acute efficacy of ESC has already been demonstrated in adolescent MDD patients in the United States, we conducted a 48-week relapse prevention study in adolescent MDD patients in Japan to evaluate the efficacy of ESC in preventing relapse and recurrence, as well as the safety of ESC.

## Methods

### Patients

This study included patients aged 12–17 years with a diagnosis of MDD or persistent depressive disorder with a current major depressive episode based on the Diagnostic and Statistical Manual of Mental Disorders, Fifth Edition (DSM-5) (American Psychiatric Association, 2013). M.I.N.I. KID version 7.0.2 (Sheehan et al., 2010) was used for diagnoses based on DSM-5. Patients with a Children's Depression Rating Scale-Revised (CDRS-R) (Poznanski et al., 1984) total score of 45 or higher and a Clinical Global Impression-Severity Scale (CGI-S) (Guy, 1976) of 4 or more were enrolled.

The main exclusion criteria were as follows: “according to DSM-5, patients with Intellectual Disability, Autism Spectrum Disorder, Attention-Deficit/Hyperactivity Disorder, Schizophrenia Spectrum and Other Psychotic Disorders, Bipolar and Related Disorders, Obsessive-Compulsive and Related Disorders, Feeding and Eating Disorders, Disruptive, Impulse-Control, and Conduct Disorders, Substance Use disorders (excluding caffeine), or Personality Disorders,” “First-degree relatives with Bipolar Disorder,” “Patients with long QT syndrome,” “Based on Columbia-Suicide Severity Rating Scale (C-SSRS), patients with a history of suicide attempt or interrupted suicide attempt within 1 year,” “patients with high risk of suicide,” “patients receiving MAO inhibitors within 2 weeks,” “patients receiving antipsychotics within 4 weeks (within 6 months for depots),” “patients receiving electroconvulsive therapy or high light therapy,” and “Pregnant, lactating, or potentially pregnant patients.”

This study was approved in advance by the Institutional Review Boards of all the participating medical institutions in Japan and was conducted in accordance with the ethical principles based on the Declaration of Helsinki and local regulatory requirements, including the Good Clinical Practice. Informed consent was obtained from proxies, and informed assent from the patients themselves was obtained.

### Study design

This study was a multicenter randomized double-blind placebo-controlled parallel group comparative study conducted at 35 Japanese medical institutions from February 2019 to April 2022.

The study consisted of a 1-week screening period, a 12-week open-label treatment period (open-label period), a 36-week double-blind treatment period (double-blind period), and a 3-week follow-up period. Patients who were considered eligible in the screening period entered the open-label period, and ESC was administered orally once daily after dinner for 12 weeks.

**FIG. 1.**
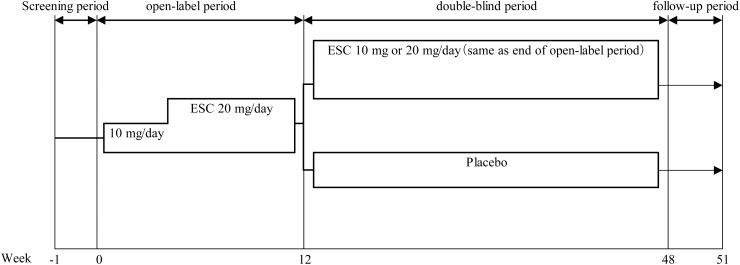
Study design. ESC was administered orally to all subjects once daily after dinner for 12 weeks. At week 12, patients who met the criteria of remission (CDRS-R total scores ≤28 and CGI-S ≤2) or response (CDRS-R total scores ≥50% improvement and CGI-S ≤2 from the start of the open-label period) continued in double-blind period and were randomly assigned to receive ESC or placebo at a 1:1 ratio. ESC or placebo was administered orally once daily after dinner for 36 weeks. ESC was administered 10 mg/day for at least 3 weeks, and the dose was allowed to increase to 20 mg/day if the investigator (or sub-investigator) judged lack of efficacy at 3, 5, 7, or 9 weeks and no AEs were found to limit the dose increase. If the investigator (or sub-investigator) judged the patient's tolerability to ESC to be problematic after dose escalation, the study visit was set as needed and the dose could be reduced until week 9. In the double-blind period, the dose at the end of the open-label period was to be administered continuously. AEs, adverse events; CDRS-R, Children's Depression Rating Scale-Revised; CGI-S, Clinical Global Impression-Severity Scale; ESC, escitalopram.

At week 12, patients who met the criteria of remission (CDRS-R total score of 28 or less and CGI-S of 2 or less) or response (CDRS-R total score of 50% or more improvement and CGI-S of 2 or less) continued in the double-blind period and were randomly assigned to receive ESC or placebo at a 1:1 ratio. ESC or placebo was administered orally once daily after dinner for 36 weeks (Fig. 1).

The dose of ESC in the open-label period was 10 or 20 mg/day the same dose for MDD patients aged 12–17 years in the United States. ESC 10 mg/day was administered for at least 3 weeks. The dose could be increased to 20 mg/day if the investigators judged lack of efficacy at 3, 5, 7, or 9 weeks and no adverse events (AEs) were found to limit the dose increase. If the investigators judged the patient's tolerability to ESC to be problematic after dose escalation, a study visit was set as needed and the dose could be reduced until week 9. The dose of ESC in the double-blind period was the same dose as at the end of the open-label period.

### Concomitant medications

Concomitant use of antidepressants other than the investigational drug was prohibited during the screening, the open-label period, and the double blind period. Only ramelteon or suvorexant was permitted as concomitant hypnotic agent, and the concomitant use of both drugs was prohibited. Patients who received hypnotic agents in the open-label period were allowed to receive the same agent in the double-blind period only at specific doses and dosages.

Other important prohibited concomitant medications included MAO inhibitors, anxiolytics, antipsychotics, antiepileptics, antiparkinsonism, antidementia, attention-deficit/hyperactivity disorder medications, lithium carbonate, and pimozide.

### Psychoeducation

Psychoeducation consisted of education on the epidemiology, causes symptoms, and course of treatment of MDD. Investigators or staff providing psychoeducation were trained before the start of the study to ensure consistency in provision. All patients received psychoeducation using a DVD prepared by the authors.

The DVD consisted of four chapters (about 10 minutes each): “If you are feeling low,” “Understanding depression,” “Treatment of depression,” and “Continuing treatment without interruption.” Psychoeducation was given during the screening period, at the start of treatment, at 3, 7, and 12 weeks, and at all prescribed visits from week 16 to 48. Patients viewed one chapter at each visit in turn, and then repeated from the first chapter after viewing the last chapter.

### Efficacy evaluation

Efficacy was assessed for relapse and CDRS-R, CGI-S, and Clinical Global Impression-Improvement (CGI-I) Scale. Relapse was defined as (1) worsening of depressive symptoms observed for at least 2 weeks during the double-blind period, and total CDRS-R score of 40 or higher, or (2) investigators deem that treatment must be changed (regardless of the CDRS-R total score). To judge relapse objectively, evaluation was performed by three independent Central Assessment Committee members.

The committee members reviewed the information on patients who discontinued treatment during the double-blind period and were considered likely to relapse based on the reason for discontinuation (e.g., lack of efficacy of the investigational drug or adverse effects), and neutrally judged relapse as to whether the patient met the definition. Patients judged to have relapsed by two or more committee members were deemed to have relapsed.

CDRS-R, CGI-S, and CGI-I scores were determined by the investigators after training to ensure uniform ratings. The primary endpoint was time to relapse in the double-blind period. In the double-blind period, secondary endpoints were relapse, change from start of double-blind period in CDRS-R total score, change from start of double-blind period in CGI-S, CGI-S worsening, CGI-I, and CGI-I improvement.

If the CGI-S increased by 2 or more from start of double-blind period, this was defined as worsening, and a CGI-I of 2 or less (“very much improved” or “much improved”) was defined as improvement. In the open-label period, secondary endpoints were change from baseline in CDRS-R total score, change from baseline in CGI-S, CGI-I, CGI-I improvement, remission, and response at week 12.

### Pharmacokinetic evaluation

Plasma concentration of ESC and S-enantiomer of DCT (S-DCT), which is the main metabolite of ESC, was measured at week 12.

### Safety evaluation

The type, incidence, and severity of AEs were assessed for safety. AEs were classified using the Medical Dictionary for Regulatory Activities (MedDRA) Ver.25.0. All AEs included in the MedDRA SMQ Suicidal/Suicide (Narrow Range) were classified as suicide-related AEs. In addition, AEs associated with self-injurious behaviors based on C-SSRS survey items (“yes” for “self-injurious behaviors without suicidal intent”) were classified as self-injury-related AEs. Laboratory test values, vital signs, body weight, and standard 12-lead ECG were also assessed at each visit.

### Statistical analysis

Based on the results of the U.S. fluoxetine study (Emslie et al., 2008), the relapse rate at week 24 in the double-blind period was assumed to be 42.9% in the ESC group and 75.0% in the placebo group, respectively, and the dropout rate was assumed to be 20% in both groups, and the risk of these events were assumed remain constant over the double-blind period.

The sample size was set at 72 patients (36 patients/group) to provide 85% power to detect superiority of the ESC group over the placebo group. The hazard ratio (HR) for relapse for the ESC group versus placebo was 2.47 under these assumptions. The number of patients in the open-label period required to ensure 72 patients continued to the double-blind period was set at 134 patients, assuming a continuation rate of 54%.

The primary analysis population used to analyze efficacy was the full analysis set in the double-blind period (FAS double-blind period). The FAS (double-blind period) included randomized patients who received at least one dose of the investigational drug in the double-blind period and who had at least one efficacy assessment after the start of the double-blind period. The primary endpoint was analyzed using the log-rank test and cox regression with treatment group as the main effect. The significance level was set at 5% (two-sided).

The FAS (open-label period) included patients who received at least one dose of the investigational drug during the open-label period and who had at least one efficacy assessment after the start of the open-label period. For secondary endpoints in the open-label period, discrete variables were analyzed by change from baseline, and binary variables were analyzed by proportions. Where secondary endpoint values were missing, they were imputed using the Last Observation Carried Forward (LOCF) method.

For secondary endpoints in the double-blind period, relapse was analyzed by Fisher's exact test and logistic regression by calculating the relapse rate. Analysis of CGI-I and change in CDRS-R total and CGI-S from start of double-blind period was carried out using analysis of covariance (ANCOVA). CGI-S worsening and CGI-I improvement were calculated as percentages (worsening rate for CGI-S and improvement rate for CGI-I) and analyzed by Fisher's exact test. If secondary endpoints were missing, they were imputed using the LOCF method. For CGI-S worsening, only the Worst Observation Carried Forward (WOCF) method was used.

## Results

### Disposition of patients and baseline characteristics

**FIG. 2.**
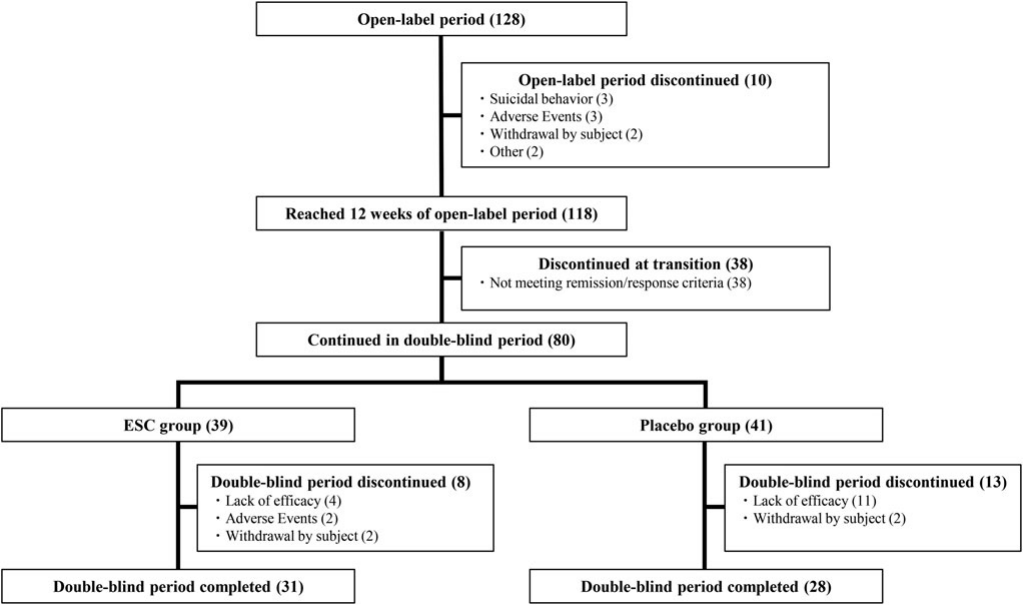
Patient disposition. All 128 patients who transitioned to open-label period were administered investigational drug and became FAS (open-label period). All of the 80 patients who continued in double-blind period and underwent randomization became FAS (double-blind period). FAS (double-blind period) was composed of 39 patients in ESC group and 41 patients in placebo group. ESC, escitalopram; FAS, full analysis set.

The study drug was administered to 128 patients who completed the screening period and entered the open-label period. Of the 118 patients who reached week 12 of the open-label period, 80 patients met remission or response criteria and were randomized to the double-blind period (39 to ESC and 41 to placebo) (Fig. 2). The most common reasons for discontinuation in the open-label period were incompatibility with the double-blind period continuation criteria of remission or response. The most common reason for discontinuation in the double-blind period were lack of efficacy of the investigational drug in both the ESC and placebo groups.

The patient demographics and baseline characteristics of FAS (open-label period) and FAS (double-blind period) are shown in Table 1. In both the open-label period and double-blind period (ESC and placebo groups), mean drug compliance was >95%.

**Table 1.** Patients' Characteristics and Baseline Characteristics

	Open-label period	Double-blind period	
	ESC (*N* = 128)	ESC (*N* = 39)	Placebo (*N* = 41)
Gender, *n* (%)			
Male	38 (29.7)	11 (28.2)	13 (31.7)
Female	90 (70.3)	28 (71.8)	28 (68.3)
Race, *n* (%)			
Asian	128 (100.0)	39 (100.0)	41 (100.0)
Black or African American	0 (0.0)	0 (0.0)	0 (0.0)
White	0 (0.0)	0 (0.0)	0 (0.0)
Other	0 (0.0)	0 (0.0)	0 (0.0)
Age (years), mean ± SD	14.9 ± 1.7	14.7 ± 1.6	15.0 ± 1.9
Weight (kg), mean ± SD	52.67 ± 10.34	54.14 ± 10.27	52.60 ± 11.15
CYP2C19, *n* (%)			
EM	104 (81.3)	32 (82.1)	32 (78.0)
PM	24 (18.8)	7 (17.9)	9 (22.0)
CDRS-R total scores (at start of drug administration), mean ± SD	65.8 ± 13.5	63.9 ± 12.7	64.2 ± 13.5
CGI-S (at start of drug administration), mean ± SD	4.7 ± 0.8	4.6 ± 0.8	4.7 ± 0.8
CDRS-R total score (at start of double-blind period), mean ± SD	—	26.7 ± 5.2	26.8 ± 5.2
CGI-S (at start of double-blind period), mean ± SD	—	1.9 ± 0.3	1.9 ± 0.4
Age at onset of depression (years), mean ± SD	13.4 ± 2.0	12.8 ± 1.8	13.5 ± 2.1
Duration from onset of depression (years), mean ± SD	1.55 ± 1.41	1.91 ± 1.55	1.52 ± 1.40
No. of depressive episodes (times), mean ± SD	1.4 ± 0.7	1.6 ± 1.0	1.4 ± 0.5
1 Time	92 (71.9)	24 (61.5)	26 (63.4)
≥2 Times	36 (28.1)	15 (38.5)	15 (36.6)
Duration of current depressive episode (months), mean ± SD	9.80 ± 9.44	9.99 ± 8.81	8.14 ± 7.35
Complications, *n* (%)	101 (78.9)	29 (74.4)	29 (70.7)
Complications of anxiety disorder	13 (10.2)	5 (12.8)	5 (12.2)
Suicidal ideation (at start of drug administration), *n* (%)^a^	32 (25.0)	7 (17.9)	11 (26.8)
Suicidal behavior (at start of drug administration), *n* (%)^b^	0 (0.0)	0 (0.0)	0 (0.0)
Adherence rate of investigational drug (%), mean ± SD	96.92 ± 4.63	95.84 ± 4.78	96.47 ± 5.82

^a^ Who answered “yes” to “(1) Wish to be dead,” “(2) Non-specific active suicidal thoughts,” “(3) Active suicidal ideation with any methods (not plan) without intent to act,” “(4) Active suicidal ideation with some intent to act, without specific plan,” or “(5) Active suicidal ideation with specific plan and intent” in the C-SSRS.

^b^ Who answered “yes” to “(6) Preparatory acts or behavior,” “(7) Aborted attempt,” “(8) Interrupted attempt,” “(9) Non-fatal suicide attempt,” or “(10) Completed suicide” in the C-SSRS.

CDRS-R, Children's Depression Rating Scale-Revised; CGI-S, Clinical Global Impression-Severity Scale; C-SSRS, Columbia-Suicide Severity Rating Scale; CYP, cytochrome P450; EM, extensive metabolizer; PM, poor metabolizer; SD, standard deviation.

### Efficacy

Results for the open-label period are shown in Table 2. The mean change from baseline in CDRS-R total score at week 12 (LOCF) was −29.3 ± 15.8 (mean ± standard deviation [SD], same hereafter). At week 12 (LOCF), the proportion of patients in remission was 43.8% (56/128 patients), response was 53.9% (69/128 patients), and remission and response was 35.9% (46/128 patients).

**Table 2.** Secondary Endpoints at the End of Open-Label Period (Full Analysis Set [Open-Label Period])

	ESC (*N* = 128)
Change in CDRS-R total score, mean ± SD	−29.3 ± 15.8
Change in CGI-S mean ± SD	−2.1 ± 1.3
CGI-I, mean ± SD	2.1 ± 0.9
CGI-I improvement rate, %	74.2
Remission, %	43.8
Response, %	53.9
Remission and response, %	35.9

Change in CDRS-R total score, change in CGI-S, CGI-I, and CGI-I improvement rate complemented missing data using LOCF method; CGI-I improvement: 2 or less in CGI-I; Remission: CDRS-R was 28 or less and CGI-S was 2 or less; Response: CDRS-R was 50% or more improvement from start of administration and CGI-S was 2 or less.

CDRS-R, Children's Depression Rating Scale-Revised; CGI-I, Clinical Global Impression-Improvement Scale; CGI-S, Clinical Global Impression-Severity Scale; ESC, escitalopram; LOCF, Last Observation Carried Forward; SD, standard deviation.

**FIG. 3.**
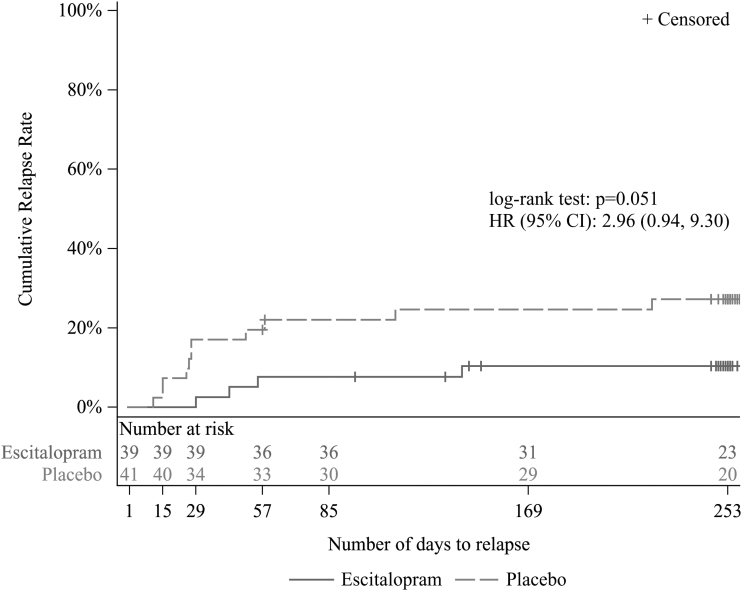
Kaplan–Meier plot for time to relapse (FAS [double-blind period]). HR was calculated as hazard ratio of placebo to ESC by Cox regression with treatment group as main effect. CI, confidence interval; FAS, full analysis set; HR, hazard ratio; ESC, escitalopram.

In the double-blind period, there was no statistically significant difference in the primary endpoint of time to relapse between the two groups (*p* = 0.051, log-rank test) (Fig. 3). In Cox proportional hazards modeling, the HR [two-sided 95% confidence interval] for relapse for the ESC group versus placebo was 2.96 [0.94–9.30]. A *post hoc* analysis, which was not planned when the study was designed, was performed using the generalized Wilcoxon test. The result was *p* = 0.044, suggesting that the ESC group may have had a longer time to relapse than the placebo group.

**FIG. 4.**
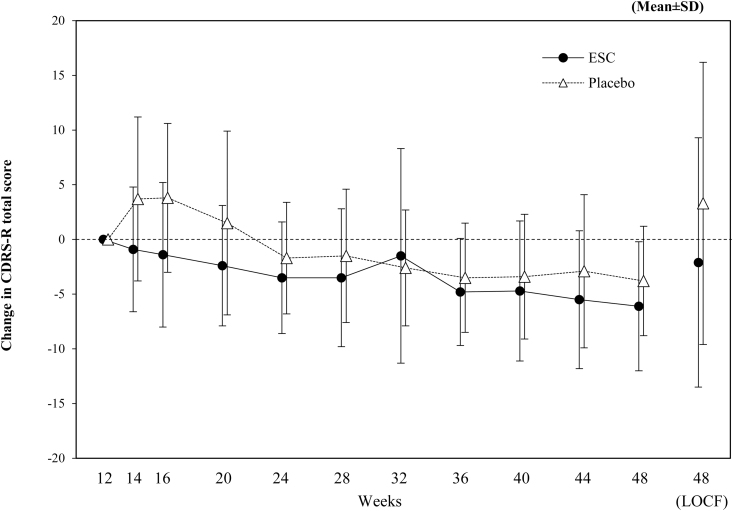
Change in CDRS-R total score from start of double-blind period (FAS [double-blind period], LOCF). Change in CDRS-R total score from start double-blind period (week 12) are shown. CDRS-R, Children's Depression Rating Scale-Revised; ESC, escitalopram; FAS, full analysis set; LOCF, Last Observation Carried Forward; SD, standard deviation.

The results of the secondary endpoints are shown in Table 3 and Figure 4. The relapse rate was 10.3% in the ESC group and 26.8% in the placebo group, with no statistically significant difference between the two groups (*p* = 0.085, Fisher's exact test). The change in CDRS-R total score from start of double-blind period to week 48 (LOCF) was −2.1 ± 11.4 in the ESC group and 3.3 ± 12.9 in the placebo group, a statistically significant lower value in the ESC group compared with placebo (*p* = 0.048, ANCOVA).

**Table 3.** Secondary Endpoints at the End of the Double-Blind Period (Full Analysis Set [Double-Blind Period])

	ESC (*N* = 39)	Placebo (*N* = 41)	Difference of groups^a^	*p* ^b^
Relapse rate, %	10.3	26.8	−16.6	0.085
Change in CDRS-R total score, mean ± SD	−2.1 ± 11.4	3.3 ± 12.9	−5.5^c^	0.048
Change in CGI-S, mean ± SD	−0.1 ± 1.0	0.4 ± 1.1	−0.5^c^	0.025
CGI-S worsening rate, %	7.7	34.1	−26.5	0.005
CGI-I, mean ± SD	1.6 ± 1.0	2.1 ± 1.1	−0.5^c^	0.030
CGI-I improvement rate, %	84.6	70.7	13.9	0.183

For change in CDRS-R total score, change in CGI-S, CGI-I, and CGI-I improvement, missing data were imputed using LOCF method; for CGI-S worsening, using WOCF method; CGI-S worsening: worsening 2 or more CGI-S from the start of double-blind period; CGI-I improvement: 2 or less in CGI-I.

^a^ ESC-placebo.

^b^ For relapse rate, CGI-S worsening rate and CGI-I improvement rate, *p* values are obtained using Fisher's exact test; for change in CGI-S and CGI-I, using ANCOVA model.

^c^ Estimates by ANCOVA model (adjusting for each score at the start of double-blind period).

CDRS-R, Children's Depression Rating Scale-Revised; CGI-I, Clinical Global Impression-Improvement Scale; CGI-S, Clinical Global Impression-Severity Scale; ESC, escitalopram; LOCF: Last Observation Carried Forward; WOCF: Worst Observation Carried Forward; SD, standard deviation.

The change in CGI-S from start of double-blind period to week 48 (LOCF) was −0.1 ± 1.0 in the ESC group and 0.4 ± 1.1 in the placebo group, a statistically significant lower value in the ESC group compared with placebo (*p* = 0.025, ANCOVA). At week 48 (WOCF), the rate of CGI-S worsening was 7.7% in the ESC group and 34.1% in the placebo group, a statistically significant lower value in the ESC group (*p* = 0.005, Fisher's exact test).

CGI-I at week 48 (LOCF) was 1.6 ± 1.0 in the ESC group and 2.1 ± 1.1 in the placebo group, a statistically significant lower value in the ESC group compared with placebo (*p* = 0.030, ANCOVA). The rate of improvement in CGI-I at week 48 (LOCF) was 84.6% in the ESC group and 70.7% in the placebo group, with no statistically significant difference between the groups (*p* = 0.183, Fisher's exact test).

### Pharmacokinetics

At week 12, plasma ESC concentrations (mean ± SD, same hereafter) were 46.82 ± 36.95 ng/mL and plasma S-DCT concentrations were 15.55 ± 6.15 ng/mL.

### Safety

The summary of AEs in this study are shown in Table 4. In the open-label period, incidence of AEs and adverse drug reactions (ADRs) were 78.9% and 60.2%, respectively. The severity of most AEs was mild or moderate. Two patients experienced severe AEs, one patient experienced serious AEs, and six patients experienced AEs leading to discontinuation. Of these, a causality relationship with the investigational drug could not be denied in two of the severe AEs, one of the serious AEs, and four of the six AEs leading to discontinuation.

**Table 4.** Summary of Adverse Events

	Open-label period	Double-blind period	
	ESC (*N* = 128),* n *(%)	ESC (*N* = 39),* n *(%)	Placebo (*N* = 41),* n *(%)
Adverse events	101 (78.9)	32 (82.1)	23 (56.1)
ADRs	77 (60.2)	9 (23.1)	9 (22.0)
Severe adverse events	2 (1.6)	2 (5.1)	0 (0.0)
Suicide attempt	2 (1.6)	0 (0.0)	0 (0.0)
Overdose	0 (0.0)	1 (2.6)	0 (0.0)
Gastroenteritis	0 (0.0)	1 (2.6)	0 (0.0)
Pneumonia	0 (0.0)	1 (2.6)	0 (0.0)
Serious adverse events	1 (0.8)	3 (7.7)	0 (0.0)
Suicide attempt	1 (0.8)	0 (0.0)	0 (0.0)
Overdose	0 (0.0)	2 (5.1)	0 (0.0)
Gastroenteritis	0 (0.0)	1 (2.6)	0 (0.0)
Pneumonia	0 (0.0)	1 (2.6)	0 (0.0)
Adverse events leading to discontinuation	6 (4.7)	2 (5.1)	0 (0.0)
Suicide attempt	3 (2.3)	0 (0.0)	0 (0.0)
Overdose	0 (0.0)	2 (5.1)	0 (0.0)
Anxiety	1 (0.8)	0 (0.0)	0 (0.0)
Suicidal behavior	1 (0.8)	0 (0.0)	0 (0.0)
Self-injurious ideation	1 (0.8)	0 (0.0)	0 (0.0)

	Follow-up period after open-label period	Follow-up period after double-blind period	
	ESC (*N* = 47),* n *(%)	ESC (*N* = 39),* n *(%)	Placebo (*N* = 41),* n *(%)
Adverse events	16 (34.0)	6 (15.4)	11 (26.8)
ADRs	3 (6.4)	2 (5.1)	3 (7.3)
Severe adverse events	1 (2.1)	1 (2.6)	1 (2.4)
Suicide attempt	1 (2.1)	0 (0.0)	0 (0.0)
Psychotic disorder due to a general medical condition	0 (0.0)	1 (2.6)	0 (0.0)
Asthenia	0 (0.0)	0 (0.0)	1 (2.4)
Serious adverse events	1 (2.1)	1 (2.6)	1 (2.4)
Suicide attempt	1 (2.1)	0 (0.0)	0 (0.0)
Psychotic disorder due to a general medical condition	0 (0.0)	1 (2.6)	0 (0.0)
Asthenia	0 (0.0)	0 (0.0)	1 (2.4)
Adverse events leading to discontinuation	0 (0.0)	0 (0.0)	0 (0.0)

All adverse events were recorded/classed according to ICH International Glossary of Pharmaceutical Glossary (MedDRA) Ver.25.0.

ADR, adverse drug reaction; ESC, escitalopram.

In the double-blind period, AE incidence was 82.1% and 56.1% in the ESC and placebo groups, respectively, and ADR incidence was 23.1% and 22.0%, respectively. The severity of most AEs was mild or moderate. In the ESC group, two patients experienced severe AEs, three patients experienced serious AEs, and two patients experienced AEs leading to discontinuation, compared with none in the placebo group. None of these AEs were causally related to ESC.

AEs occurring in at least 10% of patients in the open-label period, or in either ESC or placebo groups during the double-blind period are shown in Table 5. Headache and pyrexia occurred in at least 10% of patients in both groups in the double-blind period. In the double-blind period, there were no AEs with an incidence of at least 10% in only one group, and no ADRs with an incidence at least 5% higher in the ESC group than in the placebo group.

**Table 5.** Adverse Events Found in ≥10% (Open-Label and Double-Blind Period)

SOC PT	Open-label period	Double-blind period	
	ESC (*N* = 128),* n *(%)	ESC (*N* = 39),* n *(%)	Placebo (*N* = 41),* n *(%)
Infections and infestations			
Nasopharyngitis	10 (7.8)	8 (20.5)	4 (9.8)
Metabolism and nutrition disorders			
Decreased appetite	13 (10.2)	0 (0.0)	1 (2.4)
Nervous system disorders			
Headache	17 (13.3)	6 (15.4)	6 (14.6)
Somnolence	13 (10.2)	0 (0.0)	0 (0.0)
Gastrointestinal disorders			
Abdominal pain	3 (2.3)	4 (10.3)	1 (2.4)
Nausea	35 (27.3)	3 (7.7)	6 (14.6)
General disorders and administration site conditions			
Pyrexia	7 (5.5)	4 (10.3)	5 (12.2)
Injury, poisoning, and procedural complications			
Overdose	0 (0.0)	4 (10.3)	1 (2.4)

All adverse events were recorded/classed according to ICH International Glossary of Pharmaceutical Glossary (MedDRA) Ver.25.0. Adverse events whose incidence rate was 10% or higher were shown.

ESC, escitalopram; PT, preferred term; SOC, system organ class.

Suicide-related AEs in this study occurred in 2.3% (3/128) patients as suicide attempt and in 0.8% (1/128) patient as self-injurious ideation and suicidal behavior. Of these, two patients with suicide attempt and one patient with suicidal behavior were judged as ADRs. All of these suicide-related ADRs were observed during the open-label period or follow-up period (post-open-label period) and were serious AEs or AEs leading to discontinuation.

In addition, self-injury-related AEs occurred in 10.9% (14/128) patients as skin wound, in 2.3% (3/128) patients as overdose and in 0.8% (1/128) patient as skin abrasion, skin erosion, dermatillomania, trichotillomania, wound, and contusion. Three patients with skin wound, one patient with overdose, one patient with trichotillomania, and one patient with wound were judged as ADRs. All of these ADRs were mild and nonserious, and did not lead to discontinuation, with outcomes of recovered/resolved.

For standard 12-lead ECG, the change from baseline in QTc (QTcB and QTcF, same hereinafter; mean ± SD, same hereinafter) were 8.1 ± 17.7 and 6.4 ± 12.1 mseconds in the open-label period, and in the double-blind period were 8.4 ± 19.6 and 7.0 ± 14.4 mseconds in the ESC group, and 2.4 ± 17.3 and 1.4 ± 11.9 mseconds in the placebo group, respectively.

Although there was more than one patient with an observed value or change from baseline of QTc above the thresholds (observed value: 450 mseconds, change from baseline: 30 mseconds), the observed value was <480 mseconds and change from baseline was <60 mseconds in all patients. There were no clinically significant changes in laboratory test values, vital signs, or body weight.

## Discussion

The demographics and baseline characteristics in this study, such as gender, age, and CDRS-R total score, were generally similar to those in a placebo-controlled double-blind study of duloxetine previously conducted in Japan for adolescent MDD (Saito et al., 2022).

For the primary endpoint, time to relapse in the double-blind period, no statistically significant difference was observed between the two groups (*p* = 0.051, log-rank test). In Cox proportional hazards modeling, placebo tended to be associated with a higher risk of relapse compared with the ESC group, with a HR estimate of 2.96 for placebo.

This was numerically higher than the HR of 2.47 assumed when the study was planned, indicating a relapse-preventive effect of ESC. Compared with the log-rank test, the generalized Wilcoxon test, which is more sensitive to a difference between groups in the relapse at an early stage, was used as a *post hoc* analysis, and a statistically significant difference was found between the two groups (*p* = 0.044).

In this study, although the HR for time to relapse was numerically higher than the 2.47 (assumed when the study was planned), no statistically significant differences were observed in the log-rank test conducted as primary analysis. The reason for this was considered to be a lack of power due to the fact that the number of relapsed patients was lower than expected. Based on the U.S. fluoxetine study (Emslie et al., 2008) used for reference, 61.1% of patients in the double-blind period were expected to relapse, but in this study, only 18.8% of patients relapsed.

There were only two relapse prevention studies for adolescent depression available as a reference for relapse rates at the time this study was planned: the U.S. fluoxetine study and the U.S. citalopram study (Cheung et al., 2016). The latter included about only 10 patients per group, which was too small to use as a study design reference. Therefore, this study was designed with reference to the relapse rate of the fluoxetine study. With so few reference studies available, it was difficult to estimate the relapse rate, especially as the relapse rates in these two studies were very different.

This study was a 35-site multicenter trial, whereas the U.S. fluoxetine trial (Emslie et al., 2008) was single-center. This difference may be one of the reasons why the relapse rate in this study was lower than expected, as the placebo effect has been reported to be greater with a larger number of sites (Meister et al., 2020). Differences in baseline characteristics between the two studies may also explain the lower relapse rate in this study; even though depression severity did not differ greatly between the two studies, patients in this study had fewer complications with regard to anxiety disorders, suicidal ideation, and suicidal behavior.

This study included psychoeducation provided once a month by a trained practitioner using materials developed for this study, and this may be another reason given the reported preventive effects of psychoeducation (Prieto-Vila et al., 2021; Shimazu et al., 2011) or cognitive-behavioral therapy (CBT) (Kennard et al., 2014) against relapse/recurrence. CBT has also reported that short-term effects were comparable with placebo and statistically lower than medication, whereas the long-term effects were comparable with medication (March et al., 2007; March et al., 2004), which supports the finding that the relapse was lower than expected in this long-term study.

The results of the secondary endpoints in the open-label period were not greatly different from those in the U.S. adolescent MDD study (Esmile et al., 2009), and did not provide evidence against the efficacy of ESC.

For all secondary endpoints in the double-blind period, the ESC group showed a trend toward improvement compared with the placebo group at week 48, and four of six endpoints, including change from start of the double-blind period in CDRS-R total score, were statistically significantly different between the two groups. In addition, change in CDRS-R total score, change in CGI-S and CGI-I showed greater differences in the two groups than in the U.S. adolescent MDD study (Esmile et al., 2009), although the study design and duration were different (Table 6).

**Table 6.** Comparison of Scores with Escitalopram Adolescent Study in the United States

	This study		ESC adolescent study in the United States	
	48 Weeks (LOCF)		8 Weeks (LOCF)	
	Difference^a^	*p*	Difference^b^	*p*
Changes in CDRS-R total score	−5.5	0.048	−3.4	0.022
Changes in CGI-S	−0.5	0.025	−0.4	0.007
CGI-I	−0.5	0.03	−0.3	0.008

^a^ Mean difference between groups (ESC-placebo) of changes from baseline adjusted for start of double-blind period.

^b^ Difference between groups (ESC-placebo) in ANCOVA modeling with treatment arm and medical organization as factors and baseline values as covariates.

ANCOVA, analysis of covariance; CDRS-R, Children's Depression Rating Scale-Revised; CGI-I, Clinical Global Impression-Improvement Scale; CGI-S, Clinical Global Impression-Severity Scale; ESC, escitalopram; LOCF, Last Observation Carried Forward.

These results in the double-blind and open-label period suggest that ESC may be effective in preventing relapse.

In terms of safety profile, the AEs in this study were generally similar to those previously observed in adult clinical studies in Japan (Hirayasu, 2011a; Hirayasu, 2011b), and no issues unique to pediatric patients were observed.

The risk of suicide associated with antidepressants used to treat MDD and other psychiatric disorders in pediatric patients is of particular concern. In 2004, the FDA issued a warning to include the risk of suicidality in the Package Insert for all antidepressants used to treat children, adolescents, and young adults (Fornaro et al., 2019), and the Japanese Package Insert has similar warnings. Suicide-related serious ADRs were observed in 2 of 128 patients in this study (suicide attempt: 2 patients) and in 3 of 435 patients in Japanese adult clinical studies (intentional self-injury and intentional overdose, suicide attempt, and death [both suicide and accidental death were considered]: 1 patient each). Serious suicide-related ADRs observed in this study were events that had been reported in the adult studies, and all of serious suicide-related ADRs in these studies recovered/resolved, except for the death in the Japanese adult clinical study.

### Limitations of this study

The small number of study reports available for references in the design of this study made it difficult to estimate the number of relapses and to determine the necessary number of patients to enroll. Although this study suggested that ESC had a relapse-prevention effect, there was no statistically significant difference in the primary analysis (log-rank test for time to relapse) due to the lower relapse rate and lower power than expected. A repeat of this study that includes more patients might verify the relapse-prevention effect of ESC.

## Conclusion

There was no statistically significant difference between placebo and ESC in the main analysis. However, the results of analysis of secondary endpoints and *post hoc* analysis suggested that ESC may be protective against relapse. There were no major differences in safety or tolerability between Japanese adolescents with MDD and results from double-blind placebo-controlled parallel-group studies in Japanese adults.

## Clinical Significance

This is the first completed randomized controlled withdrawal study to evaluate the efficacy of relapse prevention and safety of antidepressant therapy in Japanese adolescents with MDD.
